# Galectin-1 and Galectin-3 mRNA expression in renal cell carcinoma

**DOI:** 10.1186/1472-6890-14-15

**Published:** 2014-04-03

**Authors:** Christoph-A von Klot, Mario W Kramer, Inga Peters, Joerg Hennenlotter, Mahmoud Abbas, Ralph Scherer, Thomas RW Herrmann, Arnulf Stenzl, Markus A Kuczyk, Juergen Serth, Axel S Merseburger

**Affiliations:** 1 Department of Urology and Urological Oncology, Hannover University Medical School, Hannover, Germany; 2 Department of Urology, Eberhard Karls University, Tuebingen, Germany; 3 Department of Pathology, Hannover University Medical School, Hannover, Germany; 4 Institute for Biometry, Hannover University Medical School, Hannover, Germany

**Keywords:** Galectin, Renal cell carcinoma, Biomarker, Prognosis

## Abstract

**Background:**

Galectins are known to regulate cell differentiation and growth as well as cell adhesion and apoptosis. Galectins have been discussed as possible prognosticators for survival in renal cell cancer (RCC) and other urological tumors. They might also play an emerging role as possible new marker-proteins for RCC. In this study, we analyzed the expression of galectin-1 and galectin-3 mRNA in order to further investigate their clinical significance in RCC.

**Methods:**

Tissue samples were obtained from 106 patients undergoing surgery for RCC. The expression of galectin-1 and galectin-3 mRNA in normal kidney and corresponding cancer tissue was analyzed using quantitative real time PCR. Differences in expression levels of paired tissue samples were assessed using paired two-sample tests. Associations of relative mRNA expression levels in tumor tissues with clinical findings were analyzed using univariate logistic regression.

**Results:**

The expression of galectin-1 (p < 0.001) and -3 (p < 0.001) mRNA were significantly higher in RCC when compared to the adjacent normal kidney tissue. For clear cell RCC, an association of male gender with higher galectin-1 and galectin-3 mRNA expression (p = 0.054, p = 0.034) was detected. For all RCCs, galectin-1 mRNA expression failed to show a significant association with advanced disease as well as a higher rate of lymph node metastases (p = 0.058, p = 0.059).

**Conclusion:**

The mRNA expression of galectin-1 and galectin-3 is significantly increased in RCC cancer tissue. The higher mRNA expression in tumor tissue of male patients raises the question of a functional connection between galectins and the higher prevalence of RCC in men. Associations with advanced disease might lead to new ways of identifying patients at higher risk of recurrent disease and might even facilitate early metastasectomy with curative intent.

## Background

RCC accounts for approximately 2–3% of all malignancies in adults with a higher incidence in males. Incidence peaks in the sixth and seventh decade of life [1,2]. A variety of risk factors for RCC, such as obesity and tobacco exposure, have been proposed in the past [1,3]. The incidence of RCC is increasing, most likely due to the wide implementation of imaging modalities, such as ultrasonography and CT-scanning [4]. The clinical course of the tumor is not or at least difficult to predict [5]. Decision making on active treatment and surveillance is therefore difficult, especially in the elderly population where aggressive surgery might put the patient at an increased peri-operative risk. Although there are no randomized controlled trials, there is evidence for a benefit of early and complete metastasectomy in patients with RCC with regard to an improved overall survival [6]. Given these difficulties of clinical decision making, there is a strong need for risk stratification in order to improve early identification of patients with recurrent disease after curative treatment. However reliable predictive biomarkers to adequately assess the hazard of treated and untreated patients with RCC are currently lacking.

Galectins are proteins of 30 kDa that can be found in the cellular nucleus, the cytosol and also in the extracellular space. They are members of the lectin family, a group of *β*-galactoside binding proteins. Lectins had previously been shown to be involved in several cellular functions, such as cell-cell interactions, cell adhesion, proliferation, angiogenesis, inflammation, fibrogenesis and apoptosis [7-10]. We previously reported on alterations of galectin expression in prostate and bladder cancer [11,12]. Out of the currently described 18 galectins, we focused our research on galectin-1 and -3 because they both had previously been correlated with the development of various malignancies [13-15]. Objective of our current study was the further characterization of galectin-1 and -3 mRNA expression in RCC. Furthermore we were looking for correlations of galectin-1 and -3 mRNA expression with clinical parameters including progression free survival.

## Methods

### Tissue sampling

Tissue from the tumor itself and adjacent corresponding tumor free renal tissue of 106 patients undergoing kidney surgery were collected between 2001 and 2005. Samples were collected at Eberhard Karls University Tuebingen. All tissue samples were obtained during the operation. Adjacent normal appearing tissues were sampled ≈0.5-2 cm distant from the margin of the primary tumor site. All samples were collected immediately after surgery, snap frozen in liquid nitrogen and stored at -80°C. Tissue was selected to include at least 75% vital tumor.

Tumor stages and histological subtypes were assessed according to the UICC 2002 issue of the TNM system [16] by two separate pathologists. Grading was based on the Fuhrman grading system [17] while histological subtypes were defined in accordance with the consensus classification of renal cell neoplasia [18]. All patients were systemic therapy-naïve and did not receive any neoadjuvant treatment before definitive surgery.

Localized RCC was defined as pT ≤ 2 without lymph node involvement or the presence of organ-metastasis and grading ≤ G2, while advanced RCCs were defined as pT ≥ 3 or lymph node positive or organ metastasis or > G2.

All data were gathered by data managers and physicians and maintained by a relational database. The approval was granted by the ethics committee (Ethics Committee of the Medical Faculty, University of Tuebingen - Germany) and informed consent was obtained from all patients prior to surgery. Patient’s clinical and histopathological parameters are summarized in Table 1.

**Table 1:** Histopathological and clinical parameters of patients with RCC

**Patient characteristics**	**All RCC all tumours**	**%**
**Total**	106	100%
**Patients**		
Age (mean; ± SD)	63.7 (11.8)	
Male	68	64.2%
Female	38	35.8%
**Histology**		
Clear cell	77	72.6%
Papillary	22	20.7%
Chromophobe	5	4.7%
Other/not classified	2	1.9%
**Stage**		
NA	3	2.8%
pT1	9	8.5%
pT1a	32	30.2%
pT1b	20	18.9%
pT2	5	4.7%
pT3	3	2.8%
pT3a	10	9.4%
pT3b	24	22.6%
pT4	0	0%
**Grade**		
G1	17	16%
G1-2	15	14.2%
G2	57	53.8%
G2-3	7	6.6%
G3	10	9.4%
LN metastasis*	11	10.4%
Visceral metastasis*	23	21.7%
**advanced/metastatic disease**		
**(pT3-4 or N+/M+/>G2)**	49	46.2%

^∗^At time of surgical intervention. SD = standard deviation, LN = lymph node, N+ = lymph node metastasis, M+ = organ metastasis.

### Patients

The mean age of all patients was 63.7 years (SD ± 11.8). The male-female ratio was 68/38 (64.2% / 35.8%). Histopathological subclassification showed 77 patients with clear cell RCC, 22 patients with papillary and five patients with chromophobe tumors; two patients had non-classified histology.

### Primary cells

For an external reference control, renal proximal tubular epithelial cells (RPTEC; Lonza (Basel, Switzerland) were cultured and prepared according to the manufacturers instructions.

### Quantitative real-time PCR analysis

Of each tissue sample two sections were stained with hematoxylin-eosin and evaluated by a pathologist. Tissue was prepared from 20 cryo sections (each 20 *μ*m). The total RNA was then extracted using TriReagent (Ambion). Conversion into single-stranded complementary DNA (cDNA) was performed using the High Capacity cDNA Reverse Transcription Kit (Applied Biosystems, Foster City, CA, USA). For quantitative real time PCR (qRT-PCR), we used the ABI 7900 Fast Sequence Detection System with universal PCR master mix and TaqMan expression assays (Applied Biosystems) according to the manufacturers instructions. The here used TaqMan assays were LGALS1 (Assay ID: Hs00355202_m1), LGALS3 (Assay ID: Hs00173587_m1), RPL13A (Hs03043885_g1), HPRT1 (Hs99999909_m1) and GUSB (Hs00939627_m1). The human RPL13A, HPRT1 and GUSB transcripts were used as endogenous controls. For biological control, we used cDNA from RPTEC primary cell transcripts. In addition, blank, no-template and no reverse transcription controls were included for each measurement. The method of Livak et al. [19] and reference ΔCt values derived from the biological reference RPTEC were used for calculation of ΔΔCt and all relative quantity values. Data were assessed using the SDS 2.3 Manager, dataAssist V1.0 software. The endogenous controls RPL13A, HPRT1 and GUSB were combined using the dataAssist software V1.0 and "arithmetic mean" as method for normalization. 106 measurements for galectin-1 expression were successfully performed; for galectin-3 one measurement did not meet the standards and was excluded. All statistics and graphics were performed using the statistical programming language R 2.15-2. For graphical analysis we used assorted differences plots, jitter plots and bean plots that were generated using R statistical software [20]. In all statistical tests the two-sided type-I-error was set to 5%. For statistical analysis of galectin mRNA expression in tumor vs. paired adjacent tissue, we used the paired *t*-test and the Wilcoxon signed-rank test. For correlation of relative expression levels with recurrence free survival we dichotomized our data according to median, mean as well as stepwise dichotomization analysis. Kaplan-Meier analysis and Cox regression was then used to evaluate the influence of mRNA expression patterns on recurrence free survival. Logistic regression was used to correlate galectin mRNA expression with clinical parameters.

## Results

**Figure 1:**
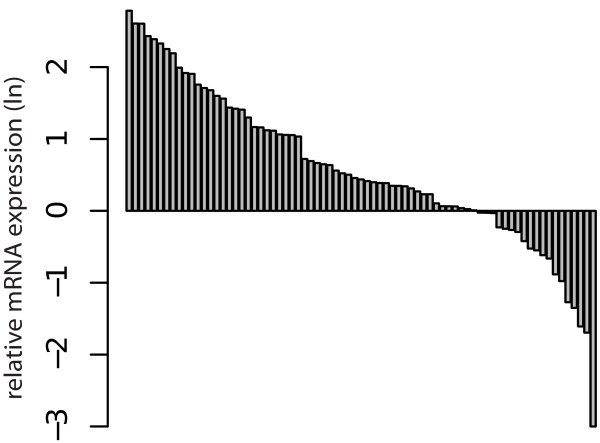
Assorted differences plot for paired analysis: Difference between relative logarithmic (ln) expressions of galectin-1 mRNA in RCC tissue compared to the surrounding normal kidney tissue (p < 0.001).

**Figure 2:**
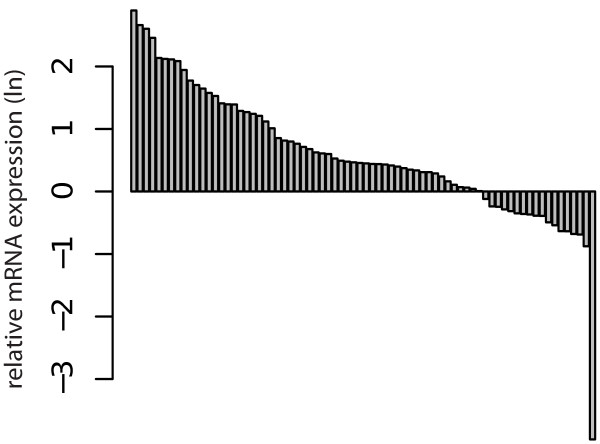
Assorted differences plot for paired analysis: Difference between relative logarithmic (ln) expressions of galectin-3 mRNA in RCC tissuecompared to the surrounding normal kidney tissue (p < 0.001).

Paired analysis of tumorous kidney tissue in comparison with adjacent microscopically normal appearing tissue was performed with 75 samples for galectin-1 and galectin-3 mRNA expression. Patient characteristics are depicted in Table 2. The mRNA expression of galectin-1 (p < 0.001, Figure 1) and galectin-3 (p < 0.001, Figure 2) were significantly higher in RCC when compared to the surrounding normal kidney tissue in paired analysis.

**Table 2:** Histopathological and clinical parameters of patients subjected to paired analysis of galectin-1 and galectin-3 expression in tumors vs. adjacent normal kidney tissue

**Patients for paired analysis**	**No.**	**%**
**Total**	75	100%
**Patients**		
Age (mean; ±SD)	67 (11.8)	
Male	46	61.3%
Female	29	38.7%
**Histology**		
Clear cell	58	77.3%
Papillary	12	16%
Chromophobe	4	5.3%
Other/notclassified	1	1.3%
**Stage**		
NA	3	4%
pT1	4	5.3%
pT1a	24	32%
pT1b	14	18.7%
pT2	4	5.3%
pT3	1	1.3%
pT3a	6	8%
pT3b	19	25.3%
pT4	0	0%
**Grade**		
G1	9	12%
G1-2	11	14.7%
G2	40	53.3%
G2-3	6	8%
G3	9	12%
LN metastasis*	7	9.3%
Visceral metastasis*	19	25.3%
**locally advanced/metastatic**		
**disease (pT3>4 and/or N/M+)**	36	48%

^∗^At time of surgical intervention. SD = standard deviation, LN = lymph node, N+ = lymph node metastasis, M+ = organ metastasis.

For galectin-1, mean mRNA expression in RCC and adjacent normal tissue was 1.57 (SD 0.86) and 0.99 (SD 0.62). For galectin-3, mean expression was -0.3 (SD 0.88) and -0.9 (SD 0.53) respectively. Relative expression levels with regard to sample and tumor characteristics are depicted in Table 3.

**Table 3:** Histopathological parameters and relative expression levels of galectin-1/3 mRNA in all patients with RCC

**Sample/pathology**	**n**	**Mean relative expression (ΔΔCt)**	**SD**
**LGALS1**	106		
Male	68	1.58	0.87
Female	38	1.37	0.91
Age (< median)	54	1.50	0.96
Age (≥ median)	52	1.48	0.81
M0	83	1.41	0.90
M+	23	1.77	0.78
N0	95	1.44	0.86
N+	11	1.97	1.03
Localized RCC*	57	1.34	0.89
Advanced RCC*	49	1.67	0.86
Grade (≤2)	89	1.46	0.85
Grade (> 2)	17	1.63	1.09
**LGALS3**	105		
Male	38	-0.41	1.04
Female	67	-0.06	0.79
Age (< median)	54	-0.25	0.97
Age (≥ median)	51	-0.12	0.82
M0	82	-0.18	0.90
M+	23	-0.23	0.92
N0	94	-0.21	0.86
N+	11	-0.001	1.23
Localized RCC*	56	-0.13	0.91
Advanced RCC*	49	-0.25	0.89
Grade (≤ 2)	88	-1.62	0.9
Grade (> 2)	17	-0.43	0.91

SD = standard deviation, N+ = lymph node metastasis, M+ = organ metastasis. ^∗^Advanced RCC was defined as pT ≥3, N1 and/or M1, or G>2.

**Figure 3:**
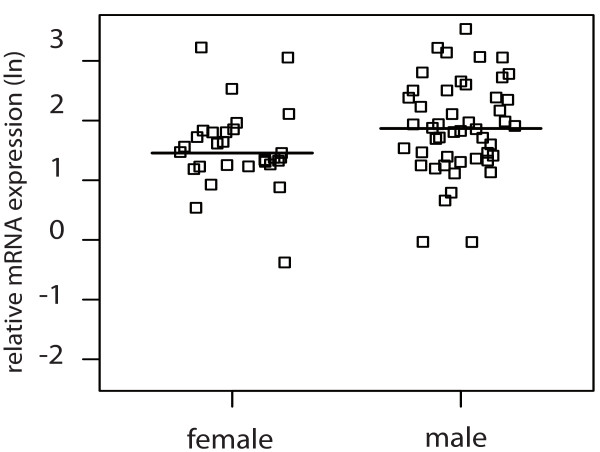
Jitter plot showing the relative logarithmic (ln) expression of galectin-1 mRNA in clear cell RCC tissue in male and female patients (p = 0.054) with the horizontal line indicating the median expression level.

**Figure 4:**
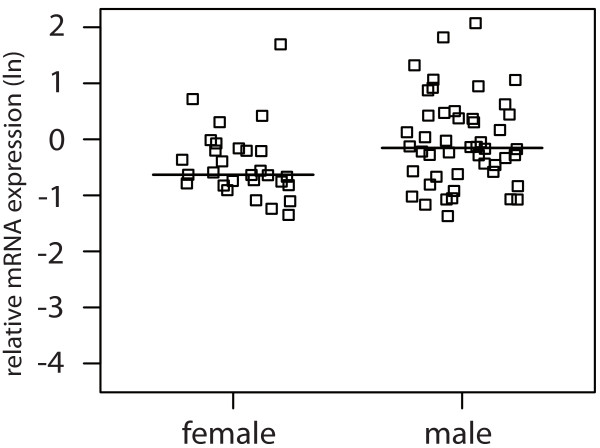
Jitter plot showing the relative logarithmic (ln) expression of galectin-3 mRNA in clear cell RCC tissue in male and female patients (p = 0.034) with the horizontal line indicating the median expression level.

For clear cell RCC, logistic regression showed an association of male gender with higher galectin-1 (p = 0.054) and -3 (p = 0.034) mRNA expression as depicted in Figure 3 and Figure 4.

These gender differences were seen exclusively in clear cell RCC tissue and did not occur in the corresponding normal tissue.

We also compared clinical parameters with the expression of galectin-1 and galectin-3. Clinical follow up was available for 43 patients. As could be expected, recurrence free survival was associated with advanced disease (p = 0.01) and metastases (p = 0.002). However no correlation between galectin-1 (p = 0.11) or galectin-3 expression (p = 0.214) and RCC recurrence after 75 month could be demonstrated in univariate analysis.

When dichotomizing our data according to the mean expression level or in stepwise dichotomization analysis, no differences with regard to recurrence free survival could be demonstrated in Cox regression or Kaplan Meier analysis.

There was no association of galectin-1 (p = 0.48) or galectin-3 (p = 0.45) mRNA expression with tumor grade.

**Figure 5:**
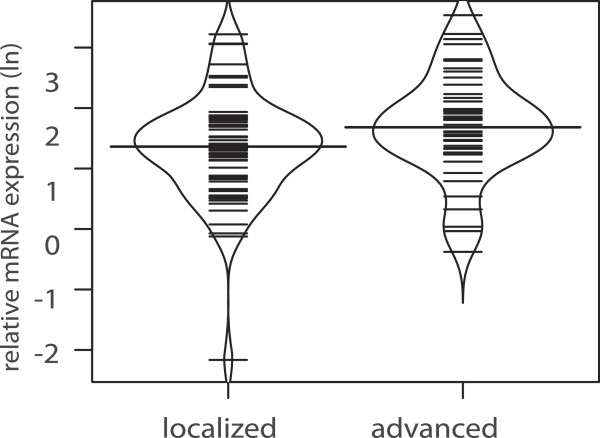
Bean plot analysis of relative logarithmic (ln) galectin-1 expression in patients with localized vs. advanced disease (p = 0.058) with the horizontal line indicating the median expression level.

**Figure 6:**
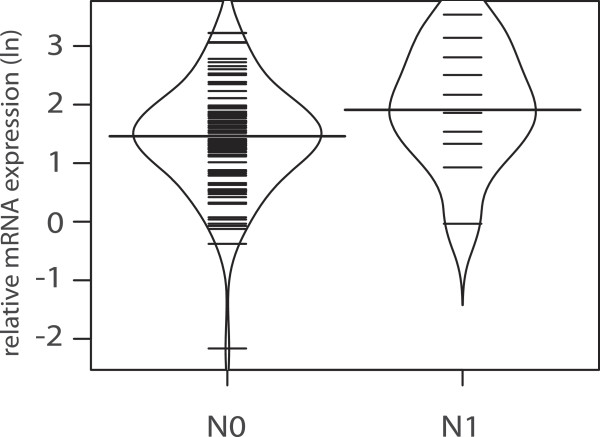
**Bean plot analysis of relative logarithmic (ln) galectin-1 expression in patients with- and without nodal involvement (p = 0.059).** The horizontal line is indicating the median expression level.

When looking at extend of disease, galectin-1 mRNA expression for all patients with RCC failed to reach the significance level with regard to advanced disease (p = 0.058, Figure 5) and lymph node metastasis (p = 0.059, Figure 6).

## Discussion

Galectin-1 had previously been shown to be elevated in a variety of cancers such as head and neck squamous cell carcinoma, thyroid cancer or hepatocellular carcinoma [13,14,21]. Moreover, the plasma levels of galectin-1 were shown to be significantly higher in RCC patients when compared to healthy controls [15]. Galectin-3 had also previously been associated with several cancer entities, such as small-cell lung cancer, gastric and colorectal cancer [22-24]. Previous studies were able to show an up-regulation of galectin-3 in RCC [25,26].

It is well known that the expression of galectin variants varies between different organ tissues [27]. Therefore we took great interest in identifying the relative galectin-1 and galectin-3 mRNA concentration by using tumor and non-tumor tissue from the same patient as reference. By doing so, we were able to eliminate any intra individual variance in distribution of galectin-1 and -3.

In contrast to our findings, previous studies implicated a possible negative correlation of galectins with regard to RCC cancer [28]. Albeit, these results were based on immunohistochemistry of proteins and not on mRNA expression analysis, they show that the physiological role of galectins is highly complicated and may vary between subtypes. Additionally, among the tumor samples of the aforementioned study a high expression of galectin-3 was initially noted that vanished with increasing stage and grade among tumors and therefore showed the reported results.

In the present study a significantly increased expression of galectin-1 and galectin-3 mRNA in RCC was shown when compared to normal adjacent renal tissue.

These findings support the hypothesis that galectin-1 and -3 may play a role in RCC progression or carcinogenesis. In this study extend of disease and lymph node involvement did not correlate with galectin-1 or galectin-3 mRNA expression.

Significance levels were narrowly missed. Others could show a significant correlation of advanced RCC and metastasis for galectin-3 using real time PCR as well as immunehistochemistry [29].

Our findings of higher galectin-1 and -3 levels in clear cell RCC tissue of male patients and the fact that neither of the two encoding genes is located on the sex chromosomes is very interesting since RCC most predominantly occurs in the male population.

With regard to a possible mechanism of action, previous data on various tumors suggest that galectin-3 may mediate invasion and the migration of cancer cells via the Wnt/ *β*-catenin signaling pathway and Akt (Protein Kinase B) phosphorylation. The proposed model describes galectin-3 to increase Akt phosphorylation, thereby increasing phosphorylation and inactivation of glycogen synthase kinase-3 *β* (GSK-3 *β*). With inactivation of GSK-3 *β*, the degradation of *β*-catenin is reduced. With increased cellular levels, *β*-Catenin can translocate to the nucleus and activate transcription after binding to transcription factors [30,31]. Others have argued that the effect of galectins in the immune T-cell response plays an important role: Higher levels of galectin-3 for instance have previously been shown to induce T-cell apoptosis, thereby providing a potential immune escape mechanism [32]. The latter findings originate from animal models, colorectal cancer research or *in vitro* studies. The impact of galectin mediated T-cell supression had not yet been fully shown in RCC [33,34].

Our findings are not without limitations. One possible issue in our evaluation might be a possible contamination of renal cell tissue with lymphocytes. Lymphatic cells are often predominantly found in or around cancerous tissue. It has been shown that the expression of galectins is upregulated in the presence of stimulated B-cells where they play a putative role in immune modulation and T-cell control [35]. Likewise immune mediated activation of T-cells is known to lead to an increased expression of galectin-1 [36]. In view of our histopathological control sections we can however exclude higher concentrations of lymphatic tissue invasion. Therefore the aforementioned confounding effects may not play an important role in this study. In addition, our data are in accordance with other studies showing equivalent elevations of galectins in cancerous tissue [26,29,37,38].

Our data on galectin-1 and -3 encourage further investigations such as the influence on survival in larger patient cohorts or the relevance of these proteins with regard to new therapeutic agents and targeted therapy. Further studies should also include the role of galectin-1 and -3 on the protein level as well as sex specific expression.

## Conclusion

To our knowledge this is the first study simultaneously correlating clinical tumor parameters with galectin-1 and -3 using real time PCR and mRNA expression patterns. The here found higher expression of galectin-1 and galectin-3 in male patients might lead to further molecular and biochemical research with regard to gender specific differences for the prevalence of RCC. The shown results regarding with advanced RCCs might lead to new ways of identifying patients at higher risk of recurrent disease and might even facilitate early metastasectomy.

## Competing interests

The authors declare that they have no competing interests.

## Authors’ contributions

CAJK, study design, interpretation of results and draft of manuscript. MWK, interpretation of results, final approval. IP, figures and interpretation of results. JH, sample selection and sample collection. MA, histopathological workup. RS, Statistical analysis. TRH, final approval. AS, final approval. MK, final approval and interpretation of results. JS, database management and quantitative real time PCR. ASM, Final approval and interpretation of results. All authors have read and approved the final manuscript.

## Pre-publication history

The pre-publication history for this paper can be accessed here:

http://www.biomedcentral.com/1472-6890/14/15/prepub

## References

[B1] Lindblad P Epidemiology of renal cell carcinoma Scand J Surg 2004 93 2 88 96 15285559 10.1177/145749690409300202

[B2] Altekruse SF Huang L Cucinelli JE McNeel TS Wells KM Oliver MN Spatial patterns of localized-stage prostate cancer incidence among white and black men in the southeastern United States, 1999-2001 Cancer Epidemiol Biomarkers Prev 2010 19 6 1460 1467 10.1158/1055-9965.EPI-09-1310 20501756 PMC2883026

[B3] Dhote R Thiounn N Debré B Vidal-Trecan G Risk factors for adult renal cell carcinoma Urol Clin North Am 2004 31 2 237 247 10.1016/j.ucl.2004.01.004 15123404

[B4] Katz DL Zheng T Holford TR Flannery J Time trends in the incidence of renal carcinoma: analysis of Connecticut Tumor Registry data, 1935-1989 Int J Cancer 1994 58 1 57 63 10.1002/ijc.2910580111 8014016

[B5] Mejean A Oudard S Thiounn N Prognostic factors of renal cell carcinoma J Urol 2003 169 3 821 827 10.1097/01.ju.0000051378.14270.2a 12576793

[B6] Kwak C Park YH Jeong CW Lee SE Ku JH Metastasectomy without systemic therapy in metastatic renal cell carcinoma: comparison with conservative treatment Urol Int 2007 79 2 145 151 10.1159/000106329 17851285

[B7] Leffler H Galectins structure and function–a synopsis Results Probl Cell Differ 2001 33 57 83 10.1007/978-3-540-46410-5_4 11190679

[B8] Hughes RC Galectins as modulators of cell adhesion Biochimie 2001 83 7 667 676 10.1016/S0300-9084(01)01289-5 11522396

[B9] Kasper M Hughes RC:Immunocytochemical evidence for a modulation of galectin 3 (Mac-2), a carbohydrate binding protein, in pulmonary fibrosis J Pathol 1996 179 3 309 316 10.1002/(SICI)1096-9896(199607)179:3<309::AID-PATH572>3.0.CO;2-D 8774488

[B10] Barondes SH Castronovo V Cooper DN Cummings RD Drickamer K Feizi T Gitt MA Hirabayashi J Hughes C Kasai K Galectins: a family of animal beta-galactoside-binding lectins Cell 1994 76 4 597 598 10.1016/0092-8674(94)90498-7 8124704

[B11] Merseburger AS Kramer MW Hennenlotter J Simon P Knapp J Hartmann JT Involvement of decreased Galectin-3 expression in the pathogenesis and progression of prostate cancer Prostate 2008 68 1 72 77 10.1002/pros.20688 18008332

[B12] Kramer MW Kuczyk MA Hennenlotter J Serth J Schilling D Stenzl A Merseburger AS Decreased expression of galectin-3 predicts tumour recurrence in pTa bladder cancer Oncol Rep 2008 20 6 1403 1408 19020721

[B13] Saussez S Lorfevre F Lequeux T Laurent G Chantrain G Vertongen F Toubeau G Decaestecker C Kiss R The determination of the levels of circulating galectin-1 and -3 in HNSCC patients could be used to monitor tumor progression and/or responses to therapy Oral Oncol 2008 44 1 86 93 10.1016/j.oraloncology.2006.12.014 17350328

[B14] Saussez S Glinoer D Chantrain G Pattou F Carnaille B André S Gabius H-J Laurent G Serum galectin-1 and galectin-3 levels in benign and malignant nodular thyroid disease Thyroid 2008 18 7 705 712 10.1089/thy.2007.0361 18630998

[B15] Kaneko N Gotoh A Okamura N Matsuo E-I Terao S Watanabe M Yamada Y Hamami G Nakamura T Ikekita M Okumura K Nishimura O Potential tumor markers of renal cell carcinoma: Alpha-Enolase for postoperative follow up, and galectin-1 and galectin-3 for primary detection Int J Urol 2012 20 5 530 535 23113677 10.1111/j.1442-2042.2012.03206.x

[B16] Sobin LH Compton CC TNM seventh edition: what’s new, what’s changed: communication from the International Union Against Cancer and the American Joint Committee on Cancer Cancer 2010 116 22 5336 5339 10.1002/cncr.25537 20665503

[B17] Fuhrman SA Lasky LC Limas C Prognostic significance of morphologic parameters in renal cell carcinoma Am J Surg Pathol 1982 6 7 655 663 10.1097/00000478-198210000-00007 7180965

[B18] Stenzl A deKernion JB Pathology, biology, and clinical staging of renal cell carcinoma Semin Oncol 1989 16 1 Suppl 1 3 11 2645654

[B19] Livak KJ Schmittgen TD Analysis of relative gene expression data using real-time quantitative PCR and the 2(-Delta Delta C(T)) Method Methods 2001 25 4 402 408 10.1006/meth.2001.1262 11846609

[B20] R Core Team R: A Language and Environment for Statistical Computing 2013 Vienna: R Foundation for Statistical Computing [ http://www.R-project.org/]

[B21] Spano D Russo R Di Maso V Rosso N Terracciano LM Roncalli M Tornillo L Capasso M Tiribelli C Iolascon A Galectin-1 and its involvement in hepatocellular carcinoma aggressiveness Mol Med 2010 16 3-4 102 115 20200618 10.2119/molmed.2009.00119 PMC2829614

[B22] Buttery R Monaghan H Salter DM Sethi T Galectin-3: differential expression between small-cell and non-small-cell lung cancer Histopathology 2004 44 4 339 344 10.1111/j.1365-2559.2004.01815.x 15049899

[B23] Baldus SE Zirbes TK Weingarten M Fromm S Glossmann J Hanisch FG Mönig SP Schröder W Flucke U Thiele J Hölscher AH Dienes HP Increased galectin-3 expression in gastric cancer: correlations with histopathological subtypes, galactosylated antigens and tumor cell proliferation Tumour Biol 2000 21 5 258 266 10.1159/000030131 10940822

[B24] Arfaoui-Toumi A Kria-Ben Mahmoud L Ben Hmida M Khalfallah MT Regaya-Mzabi S Bouraoui S Implication of the Galectin-3 in colorectal cancer development (about 325 Tunisian patients) Bull Cancer 2010 97 2 E1 E8 20080461 10.1684/bdc.2010.1032

[B25] Young AN Amin MB Moreno CS Lim SD Cohen C Petros JA Marshall FF Neish AS Expression profiling of renal epithelial neoplasms: a method for tumor classification and discovery of diagnostic molecular markers Am J Pathol 2001 158 5 1639 1651 10.1016/S0002-9440(10)64120-X 11337362 PMC1891957

[B26] François C van Velthoven R De Lathouwer O Moreno C Peltier A Kaltner H Salmon I Gabius HJ Danguy A Decaestecker C Kiss R Galectin-1 and galectin-3 binding pattern expression in renal cell carcinomas Am J Clin Pathol 1999 112 2 194 203 10439799 10.1093/ajcp/112.2.194

[B27] Kim H Lee J Hyun JW Park JW Joo HG Shin T Expression and immunohistochemical localization of galectin-3 in various mouse tissues Cell Biol Int 2007 31 7 655 662 10.1016/j.cellbi.2006.11.036 17222570

[B28] Merseburger AS Kramer MW Hennenlotter J Serth J Kruck S Gracia A Stenzl A Kuczyk MA Loss of galectin-3 expression correlates with clear cell renal carcinoma progression and reduced survival World J Urol 2008 26 6 637 642 10.1007/s00345-008-0294-8 18594826

[B29] Sakaki M Fukumori T Fukawa T Elsamman E Shiirevnyamba A Nakatsuji H Kanayama H-O Clinical significance of Galectin-3 in clear cell renal cell carcinoma J Med Invest 2010 57 1-2 152 157 20299755 10.2152/jmi.57.152

[B30] Zhang D Chen Z-G Liu S-H Dong Z-Q Dalin M Bao S-S Hu Y-W Wei F-C Galectin-3 gene silencing inhibits migration and invasion of human tongue cancer cells in vitro via downregulating beta-catenin Acta Pharmacol Sin 2013 34 1 176 184 10.1038/aps.2012.150 23103626 PMC4086502

[B31] Song S Mazurek N Liu C Sun Y Ding QQ Liu K Hung M-C Bresalier RS Galectin-3 mediates nuclear beta-catenin accumulation and Wnt signaling in human colon cancer cells by regulation of glycogen synthase kinase-3beta activity Cancer Res 2009 69 4 1343 1349 10.1158/0008-5472.CAN-08-4153 19190323 PMC2990400

[B32] Fukumori T Takenaka Y Yoshii T Kim H-RC Hogan V Inohara H Kagawa S Raz A CD29 and CD7 mediate galectin-3-induced type II T-cell apoptosis Cancer Res 2003 63 23 8302 8311 14678989

[B33] Hsu DK Chen HY Liu FT Galectin-3 regulates T-cell functions Immunol Rev 2009 230 1 114 127 10.1111/j.1600-065X.2009.00798.x 19594632

[B34] Peng W Wang HY Miyahara Y Peng G Wang RF Tumor-associated galectin-3 modulates the function of tumor-reactive T cells Cancer Res 2008 68 17 7228 7236 10.1158/0008-5472.CAN-08-1245 18757439 PMC3181121

[B35] Zuñiga E Rabinovich GA Iglesias MM Gruppi A Regulated expression of galectin-1 during B-cell activation and implications for T-cell apoptosis J Leukoc Biol 2001 70 1 73 79 11435488

[B36] Fuertes MB Molinero LL Toscano MA Ilarregui JM Rubinstein N Fainboim L Zwirner NW Rabinovich GA Regulated expression of galectin-1 during T-cell activation involves Lck and Fyn kinases and signaling through MEK1/ERK, p38 MAP kinase and p70S6 kinase Mol Cell Biochem 2004 267 1–2 177 185 15663199 10.1023/b:mcbi.0000049376.50242.7f

[B37] Masui O White NMA DeSouza LV Krakovska O Matta A Metias S Khalil B Romaschin AD Honey RJ Stewart R Pace K Bjarnason GA Siu KWM Yousef GM Quantitative proteomic analysis in metastatic renal cell carcinoma reveals a unique set of proteins with potential prognostic significance Mol Cell Proteomics 2013 12 1 132 144 10.1074/mcp.M112.020701 23082029 PMC3536894

[B38] Straube T Elli AF Greb C Hegele A Elsässer H-P Delacour D Jacob R Changes in the expression and subcellular distribution of galectin-3 in clear cell renal cell carcinoma J Exp Clin Cancer Res 2011 30 89 10.1186/1756-9966-30-89 21958686 PMC3220637

